# Clinical application of the anti‐human telomerase reverse transcriptase (hTERT) antibody (SCD‐A7) immunocytochemistry in liquid‐based urine cytology: A prospective, single institute study

**DOI:** 10.1002/cam4.5767

**Published:** 2023-03-14

**Authors:** Ji Hye Moon, Ilias P. Nikas, Kyung Chul Moon, Bohyun Kim, Han Suk Ryu

**Affiliations:** ^1^ Department of Pathology, Kangbuk Samsung Hospital Sungkyunkwan University School of Medicine Seoul Republic of Korea; ^2^ School of Medicine European University Cyprus Nicosia Cyprus; ^3^ Department of Pathology Seoul National University Hospital Seoul Republic of Korea; ^4^ Department of Pathology Seoul National University College of Medicine Seoul Republic of Korea; ^5^ Department of Pathology, Konkuk University Medical Center Konkuk University School of Medicine Seoul South Korea; ^6^ Center for Medical Innovation, Biomedical Research Institute Seoul National University Hospital Seoul Republic of Korea

**Keywords:** biomarker, cytology, immunocytochemistry, telomerase reverse transcriptase, urine, urothelial carcinoma

## Abstract

**Objectives:**

Urine cytology is the most widely used noninvasive screening tool for urothelial carcinoma diagnosis and surveillance. Although highly specific, urine cytology exhibits suboptimal sensitivity. This study aimed to determine whether hTERT immunocytochemistry (ICC) could be applicable as an ancillary test in routine cytology practice.

**Methods:**

A total of 561 urinary tract samples were initially screened in this study. All of them were prepared using SurePath liquid‐based cytology (LBC), while additional LBC slides were made and subsequently used for hTERT (SCD‐A7) ICC.

**Results:**

From the 561 samples screened, 337 were finally analyzed, all having an adequate cellularity and available follow‐up histology. The hTERT ICC‐positive rate was 95.9% (*n* = 208/217), 96% (*n* = 24/25), and 100% (*n* = 4/4) in cytology samples with high‐grade urothelial carcinoma, carcinoma in situ, and low‐grade urothelial carcinoma subsequent histology. Among the 64 atypical cytology cases histologically confirmed as urothelial carcinomas, 92.2% (*n* = 59/64) were immunoreactive to hTERT, whereas the two histologically benign cases were ICC‐negative. 87/90 (96.7%) of the cytology cases confirmed to be benign in follow‐up were hTERT‐negative. The overall sensitivity and specificity of hTERT ICC were 96.3% and 98.8%, respectively (AUROC = 0.963; 95% CI = 0.960–0.967).

**Conclusions:**

The hTERT ICC test exhibited consistent and intense staining in malignant urothelial cells, suggesting its value as an ancillary test in liquid‐based urine cytology.

## Graphical abstract

In this study, we describe a prospective validation study to evaluate the reliability of hTERT immunocytochemistry in urine cytology using liquid‐based preparation. The results showed constant and intense staining in urothelial carcinoma and high sensitivity and specificity in the cohort.

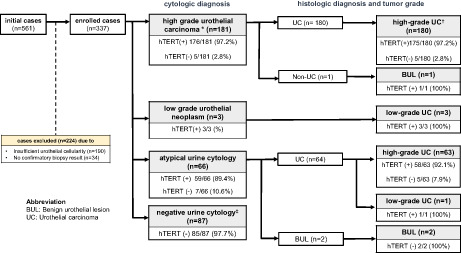

## 1 INTRODUCTION

Urine cytology is a widely used, first‐line screening tool for the diagnosis of urothelial carcinoma. Even though the diagnostic accuracy of this test has been relatively high for detecting high‐grade urothelial carcinoma, reported sensitivity ranges from 26.3% to 88%, depending on the type of sample collection.^1^, ^2^ Especially, urine cytology has a lower accuracy rate and interobserver variability^3^, ^4^ for the indeterminate diagnosis of “atypical urothelial cells” (AUC), which encompasses a wide range of urothelial lesions from non‐neoplastic conditions to malignancy.^5^

As the atypical category could subsequently reveal urothelial carcinoma in follow‐up cytology or surgical pathology, this leads to confusion of clinicians on how to interpret this result and manage patients with atypical urine cytology interpretations.^5^, ^6^, ^7^ Therefore, urine‐based molecular tests, such as the UroVysion fluorescence in situ hybridization (FISH) assay (Vysis), Nuclear matrix protein (NMP) 22 test (Matritech), and ImmunoCyt test (DiagnoCure), have been approved as ancillary tests in the diagnosis and surveillance of urothelial malignancies. However, the overall sensitivity and specificity of these tests are suboptimal, especially when they lack a simultaneous cytological assessment, while the latter is still considered the gold standard in these settings.^8^ For that reason, immunostaining of cells has been introduced in urine cytology to identify potential biomarkers, with the goal to enhance its overall diagnostic performance.^9^, ^10^

Recently, immunocytochemistry (ICC) with the anti‐hTERT (human telomerase reverse transcriptase) monoclonal antibody (SCD‐A7, INOVIQ Ltd.) has been approved by the US Food and Drug Administration (FDA) for the detection and surveillance of bladder cancer in urine cytology. hTERT is a highly conserved catalytic subunit of the telomerase holoenzyme, which is responsible for maintaining the DNA sequence at the end of the chromosome, reextending it to its normal length after each cell division. Telomerase is known to be activated, at a low level, only in a small subset of normal stem cells, whereas it has been shown to be augmented in many cancer cell types, maintaining their telomere length and inducing their immortality.^11^, ^12^, ^13^ Being closely related to telomerase, hTERT high expression is a sufficient surrogate marker of enhanced telomerase activity.^14^ Increased transcription of hTERT has been detected in primary and recurred bladder cancer.^15^ In this context, there have been a few previous attempts to detect hTERT in urine or urinary bladder tissue samples.^16^, ^17^, ^18^

In this article, we describe a prospective validation study to evaluate the reliability of hTERT ICC in urine cytology. Also, we aimed to determine whether this biomarker could effectively triage the atypical urine cytology cases, by identifying the ones harboring urothelial carcinoma in follow‐up histology.

## 2 MATERIALS AND METHODS

### 2.1 Case selection

This prospective study initially reviewed 561 urine cytology cases collected at Seoul National University Hospital (SNUH) between December 2019 and May 2022. The urine samples were mainly voided (531 out of 561), whereas 5.3% of them (30 out of 561) were catheterized and washings. The cytologic diagnosis was rendered by two experienced cytopathologists (J.H.M and H.S.R). Among the reviewed cases, a total of 413 were cytologically atypical, suspicious, or malignant. The rest 148 contained normal urothelium without any evidence of carcinoma from both cytologic evaluation and the clinical information from each patient's electronic medical records. The original cytology diagnoses were reclassified for this study according to the Paris system for Reporting Urinary Cytology^19^ as: (1) negative for high‐grade urothelial carcinoma (NHGUC), (2) atypical urothelial cells (AUC), (3) suspicious for high‐grade urothelial carcinoma (SHGUC), (4) high‐grade urothelial carcinoma (HGUC), and (5) low‐grade urothelial neoplasm (LGUN). The study protocol was reviewed and approved by the institutional review board of the Seoul National University Hospital (IRB no. 1907‐183‐1051). The waiver of informed consent was granted by the IRB.

### 2.2 Cytologic preparations and ICC for hTERT

Samples were prepared with the BD SurePath (Becton, Dickinson and Company) liquid‐based cytology (LBC) protocol, followed by Papanicolaou staining.^20^ Following the routine diagnostic procedure, residual SurePath urine samples were processed on a separate slide for hTERT ICC. The latter was performed according to the manufacturer's guidelines. Briefly, slides were fixed in 1:1 acetone and methanol for 10 min at room temperature, and then placed into 10% neutral buffered formalin for 2 min at room temperature. They were then rinsed into a Ventana reaction buffer and kept “wet” during transfer to the ICC lab. Antigen retrieval was carried out at 95°C for 32 min using the Benchmark Ultra CC1 (Roche Diagnostics). Anti‐hTERT Antibody (SCD‐A7) was diluted (1:30) in Ventana Antibody Diluent with Casein (Roche Diagnostics) and incubated at 37°C for 32 min. The OptiView DAB detection system was used. Hematoxylin II was incubated for 16 min, while the bluing reagent was incubated for 4 min. The anti‐hTERT immunostaining was localized into the nucleus.

For ICC assessment, samples containing less than 15 urothelial cells (either isolated and/or within clusters) were deemed insufficient cellularity and excluded from subsequent analysis. The lymphocytes, common in patients presenting with hematuria or tumor, were used as an internal positive control. Immunoreaction of hTERT in two or more cells, with an increased nucleus‐to‐cytoplasmic (N/C) ratio (>0.5) and/or hyperchromatic nucleus, was considered as a positive ICC result.

### 2.3 Statistical analysis

The receiver‐operating characteristic (ROC) curve analysis was used to assess the diagnostic efficacy of hTERT in differentiating malignant from benign urinary cytology cases. The sensitivity (SN), specificity (SP), diagnostic accuracy, and area under the ROC curve (AUROC) of the hTERT ICC with 95% confidence interval (CI) were also evaluated. The analysis was performed with R, version 4.1.2 (R Foundation for Statistical Computing) with the packages “caret”^22^ and “pROC.”^23^

## 3 RESULTS

### 3.1 Patient characteristics

**FIGURE 1:**
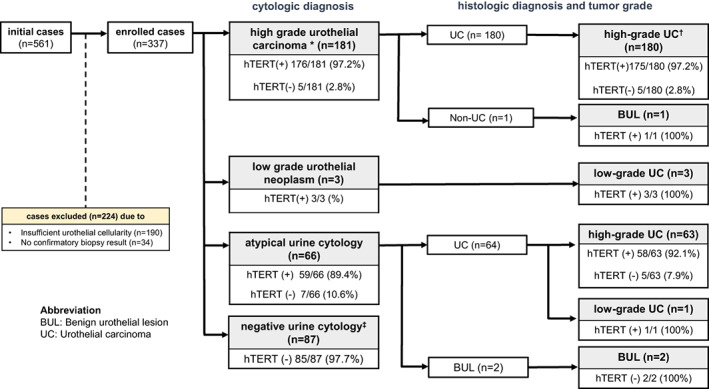
Flowchart of our study showing the number of urinary cytology samples processed in each step, also the hTERT ICC results, stratified according to the cytologic and their paired histologic diagnoses. *The cytologic diagnostic categories, “high‐grade urothelial carcinoma (HGUC)” and “suspicious for high‐grade urothelial carcinoma (SHGUC)” are included, according to the Paris System for Reporting Urinary Cytology. ^†^The “high‐grade UC” encompasses high‐grade urothelial carcinoma, carcinoma in situ and adenocarcinoma cases. ^‡^Negative for high‐grade urothelial carcinoma (NHGUC). hTERT, human telomerase reverse transcriptase; ICC, immunocytochemistry; UC, urothelial carcinoma.

A total of 561 samples were collected during the study period. In the first screening of hTERT ICC slides, 190 and 34 samples were excluded due to insufficient cellularity and the absence of follow‐up results, respectively. Finally, a cohort of 337 samples composed of HGUCs and SHGUCs (*n* = 181), AUCs (*n* = 66), LGUNs (*n* = 3), and NHGUCs (*n* = 87) was enrolled in the study (Figure 1). All 337 samples analyzed had sufficient cellularity and available follow‐up result for comparison. The demographic findings and histologic diagnoses of these cases are described in Table 1. The mean age of the patients included was 71.8 years (range, 45–91 years), while the cohort consisted of 266 males and 71 females (male:female ratio = 3.7:1). The follow‐up histological diagnoses were as follows: 99 (29.4%) noninvasive papillary urothelial carcinomas; 25 (7.4%) carcinomas in situ (CIS); 86 (25.5%) invasive urothelial carcinomas, submucosal invasive; 36 (10.7%) invasive urothelial carcinomas, muscle invasive; and 1 (0.3%) adenocarcinoma of the urinary bladder. In addition, 90 cases (26.7%) were found to be benign (Table 1).

**TABLE 1:** Demographic findings and histologic diagnoses of the cases included in this study.

Variables	*N* (%)
Age	
Mean (range)	71.8 (45–91)
Sex (%)	
Male	266 (78.9)
Female	71 (21.1)
Histologic diagnosis	
Noninvasive papillary carcinoma, high‐/low‐grade	99 (29.4%)
Carcinoma in situ	25 (7.4%)
Invasive urothelial carcinoma, submucosal invasive	86 (25.5%)
Invasive urothelial carcinoma, muscle invasive	36 (10.7%)
Adenocarcinoma of urinary bladder, muscle invasive	1 (0.3%)
Benign urothelial lesion	90 (26.7%)
Tumor grade in urothelial carcinoma	
High‐grade^a^/Carcinoma in situ	243
Low‐grade	4
Total	337

^a^ Includes adenocarcinoma of the urinary bladder.

### 3.2 Negative immunoreactivity of hTERT in most NHGUC cases

**FIGURE 2:**
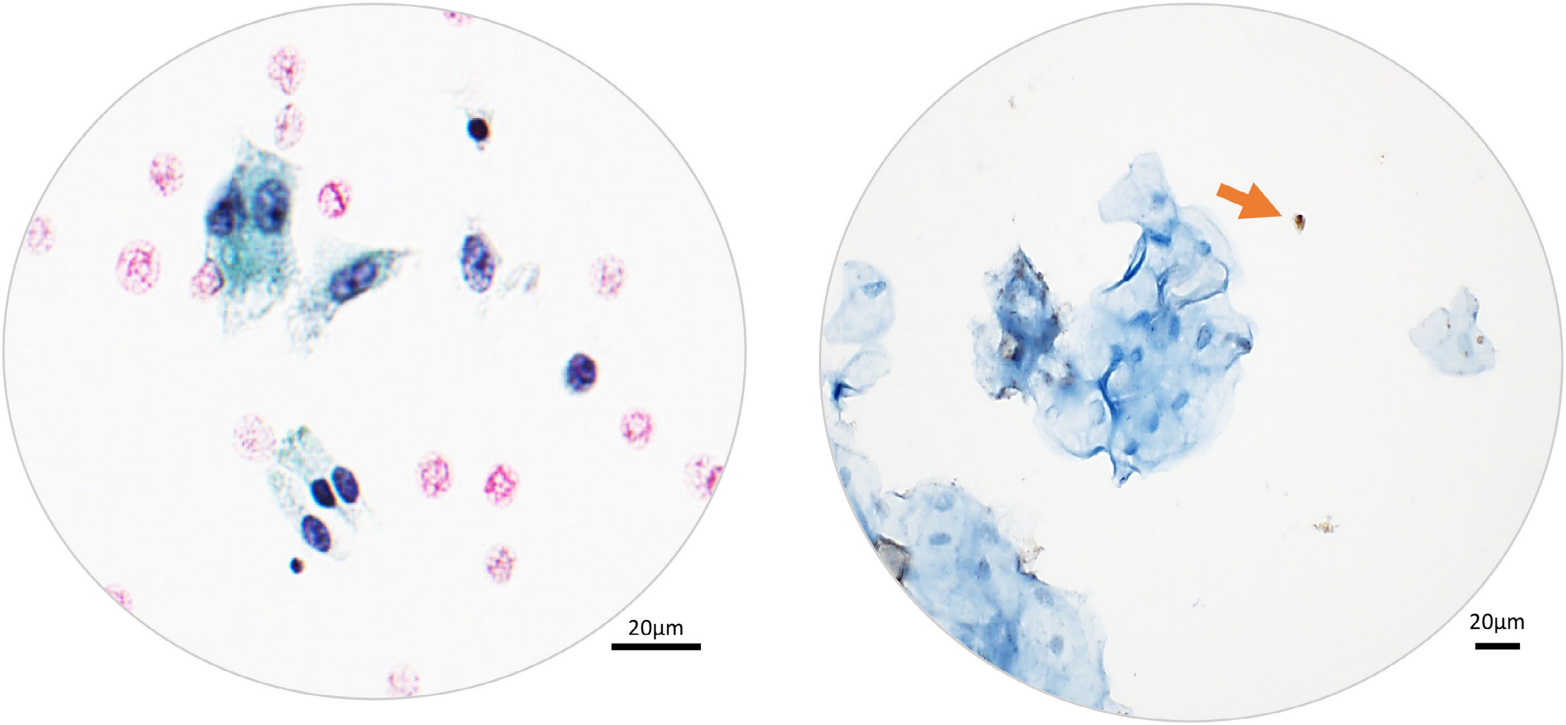
Negative hTERT immunocytochemistry (ICC) in benign cellular elements found in urine cytology. Immunostaining of lymphocytes (arrow) was used as a positive internal control (Left: Papanicolaou stain, ×400. Right: hTERT ICC, ×400).

Eighty‐seven urine cytology cases interpreted as NHGUC and without a clinical history of urinary disease were selected to be stained with hTERT (Table 2). Lymphocytes were regarded as a positive internal control. Whereas hTERT was strongly stained in lymphocytes, most benign urothelial cells (including umbrella cells) showed no or weak nuclear immunoreactivity (Figure 2). As a result, just 2/87 NHGUC cases (2.3%) exhibited strong hTERT immunopositivity. Similarly, 96.7% (*n* = 87/90) of the cases confirmed to be benign during follow‐up were hTERT‐negative (Table 2).

**TABLE 2:** Results of hTERT ICC in the final urinary cytology cohort analyzed, composed of 337 cases.

Variables	hTERT ICC		Total
	Positive (%)	Negative (%)	
Cytologic diagnosis			
High‐grade malignancy^a^	176 (97.2)	5 (2.8)	181
Atypical urothelial cells	59 (89.4)	7 (10.6)	66
Low‐grade urothelial neoplasm	3 (100.0)	0	3
NHGUC	2 (2.3)	85 (97.7)	87
Total			337
Confirmatory diagnosis on follow‐up histology			
Malignancy			
HG urothelial carcinoma	208 (95.9)	9 (4.1)	217
Adenocarcinoma of urinary bladder	1 (100)	0	1
LG urothelial carcinoma	4 (100)	0	4
Urothelial CIS	24 (96.0)	1 (4.0)	25
Benign	3 (3.3)	87 (96.7)	90
Total			337

Abbreviations: CIS, carcinoma in situ; HG, high‐grade; ICC, immunocytochemistry; LG, low‐grade; NHGUC, negative for high‐grade urothelial carcinoma.

a Includes the Paris System cytology categories “High‐grade urothelial carcinoma” and “Suspicious for high‐grade urothelial carcinoma”.

### 3.3 Positive immunoreactivity of hTERT in urothelial carcinoma

**FIGURE 3:**
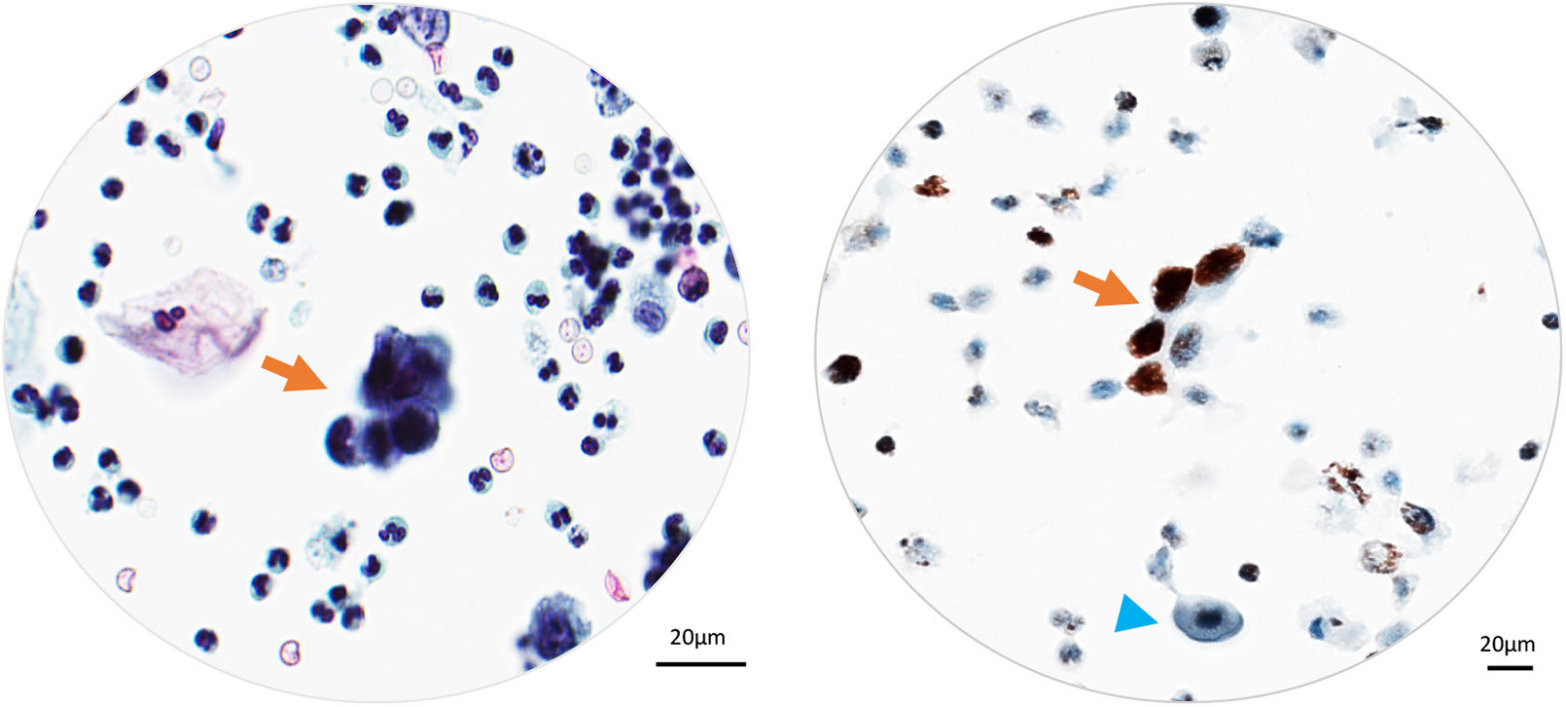
Positive hTERT staining pattern in a malignant urine sample with sufficient internal controls. Benign urothelial cells were hTERT‐negative (blue arrowhead), whereas malignant urothelial cells were hTERT‐positive (orange arrow). Left: Papanicolaou stain, ×400. Right: hTERT ICC, ×400.

We enrolled 181 SHGUCs and HGUCs with sufficient cellularity for hTERT ICC, 180 of which were also histologically confirmed as urothelial carcinomas. Among these 180 cases, 175 (97.2%) showed strong positive hTERT immunoreactivity in carcinoma cells (Figure 1). By contrast, adjacent normal urothelial cells were hTERT‐negative or weakly positive (Figure 3).

**FIGURE 4:**
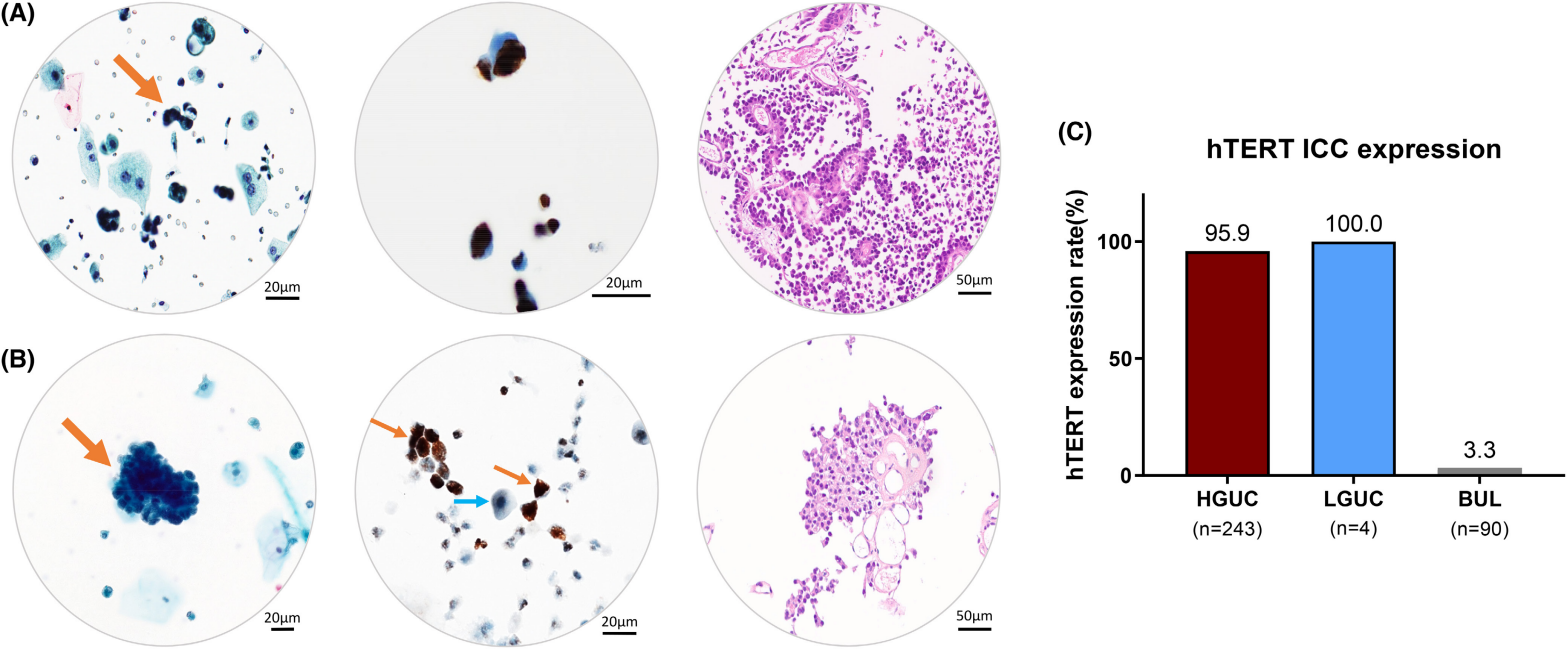
Matched photomicrographs of urothelial carcinoma classified as (A) high‐grade and (B) low‐grade urothelial cell carcinoma (orange arrow, malignant cells; blue arrow, benign urothelial cells). The photomicrographs are in the following order: Papanicolaou stain, ×400; hTERT ICC stain, ×400; transurethral biopsy, ×200, from left to right. (C) Positive rate of hTERT ICC, according to WHO/ISUP tumor grade. BUL benign urothelial lesion; HGUC, high‐grade urothelial carcinoma; LGUC, low‐grade urothelial carcinoma.

We further evaluated the hTERT ICC expression of our cases in relation to their follow‐up histologic diagnosis, according to the 4th edition of the WHO classification.^27^ From the 247 cases diagnosed as malignant during follow‐up histology, 243 were classified as high‐grade (98.4%), including 217 high‐grade urothelial carcinomas, 25 CIS, and one bladder adenocarcinoma (Tables 1 and 2). hTERT protein was overexpressed in 233/243 (95.9%, Figure 4A and 4C) of these neoplasms. The rest four cases (1.6%), which were classified as low‐grade urothelial carcinomas, showed 100% positive hTERT immunoreactivity (4/4, Figure 4B,C).

### 3.4 hTERT protein expression pattern in AUC

**FIGURE 5:**
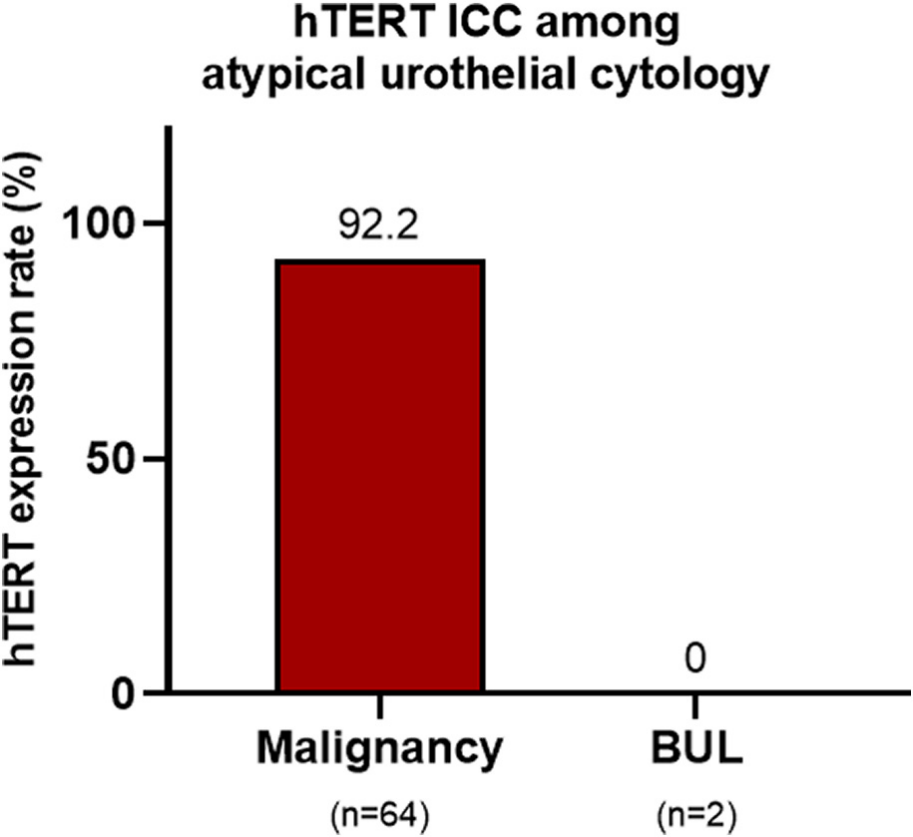
Results of hTERT immunocytochemistry (ICC) in the subgroup comprising AUC cytology cases. AUC, atypical urothelial cells; BUL, benign urothelial lesion.

To evaluate the diagnostic efficacy of hTERT ICC in detecting urothelial carcinoma in the subgroup comprising atypical cytology samples, 187 urine LBC samples with an AUC interpretation were examined; 98 with inadequate cellularity were excluded. Of the remaining 89 cases, matched histologic diagnosis was available in 66 cases, which were included in the final analysis (Table 2); 64 of them were histologically confirmed as urothelial carcinoma, whereas two as benign. Among the 64 AUC cases confirmed as malignant in follow‐up histology, 59 (92.2%) were hTERT‐positive (Figure 5), whereas five (7.8%) were hTERT‐negative (Table 3). Both AUC samples, eventually turned out to be benign in their matched histology, were found to be hTERT‐negative.

**TABLE 3:** Results of hTERT ICC in the “atypical urothelial cells” (AUC) subgroup of our study, composed of 66 cases.

Confirmatory histologic diagnosis	hTERT ICC result	
	Positive	Negative
Malignant	59 (92.2%)	5 (7.8%)
Adenocarcinoma	1	0
CIS	4	0
UC LG	1	0
UC HG, NIP	22	1
UC HG, stromal invasive	20	1
UC HG, muscle invasive	11	3
Benign	0	2 (100%)

Abbreviations: CIS, carcinoma in situ; NIP, noninvasive papillary; UC HG, urothelial carcinoma, high‐grade; UC LG, urothelial carcinoma, low‐grade.

### 3.5 Correlation of hTERT expression between malignant and benign urine cytology

**FIGURE 6:**
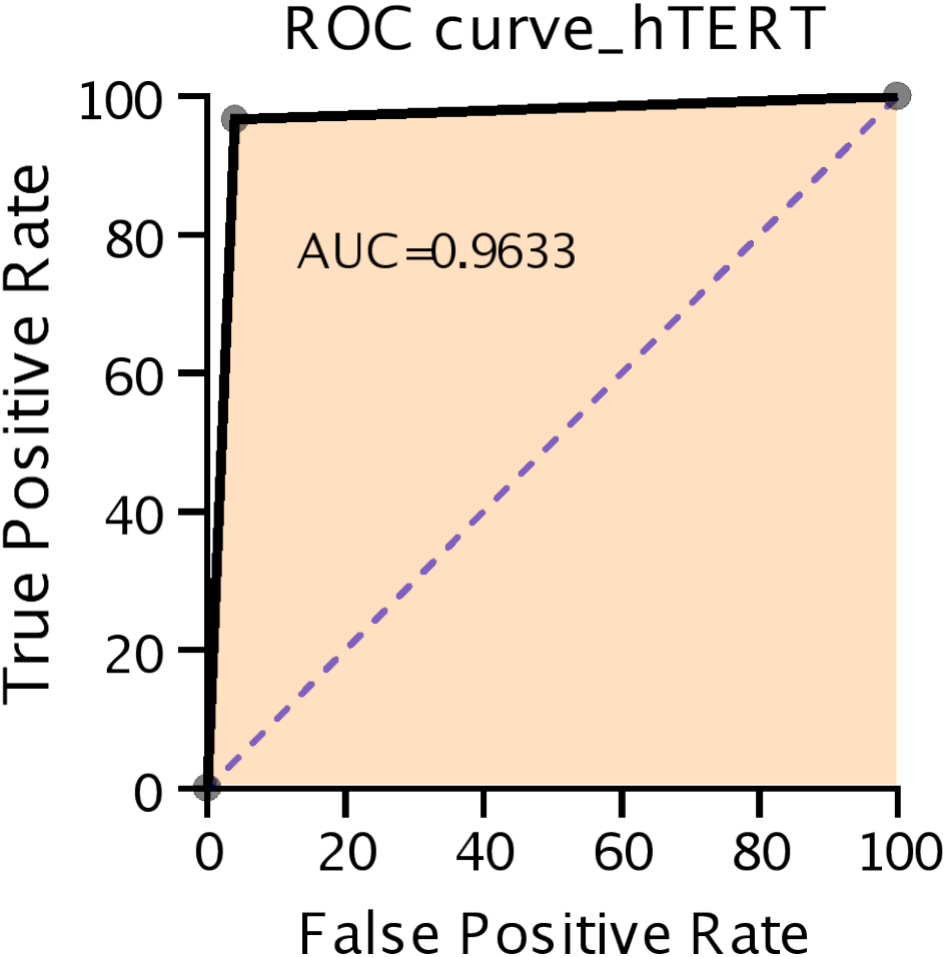
ROC curve analysis of hTERT immunocytochemistry in our study. AUROC value was 0.963 (95% CI: 0.960–0.967). AUROC, area under the receiver‐operating curve; CI, confidence interval; ROC, receiver‐operating curve.

The diagnostic performance of hTERT ICC to detect the presence of cancer was analyzed (Table 4). The assessment of the overall diagnostic accuracy of hTERT ICC in our LBC cohort showed the following results: 96.3% SN, 98.8% SP, and 97.0% (95% CI: 94.5–98.5) accuracy. The hTERT SN in the subgroup composed of SHGUC and HGUC cytology cases was 97.2%, while the accuracy was 96.7% (95% CI: 93.0–98.7). The SN of hTERT to detect cancer in the subgroup comprising AUC cases was 92.2%. SP could not be calculated, as all samples with benign histological follow‐up were hTERT‐negative in the subgroup. The ROC curve of hTERT ICC in total of 337 cases showed an AUROC value of 0.963 (95% confidence interval [CI: 0.960–0.967], Figure 6).

**TABLE 4:** Sensitivity and specificity of hTERT ICC in the urine cytology cases analyzed in our study.

	Sensitivity (%)	Specificity (%)	Accuracy, % (95% CI)
All cases (*n* = 337)	96.3	98.8	97.0 (94.5–98.5)
High‐grade urothelial carcinoma cytology^a^ (*n* = 184)	97.2	0	96.7 (93.0–98.7)
Atypical urothelial cytology (*n* = 66)	92.2	100.0	92.4 (83.2–97.5)

Abbreviations: CI, confidence interval; ICC, immunocytochemistry.

a Includes the Paris System cytology categories “Suspicious for high‐grade urothelial carcinoma (SHGUC)” and “High‐grade urothelial carcinoma (HGUC).”.

## 4 DISCUSSION

In this study, we demonstrated the value of hTERT as a novel diagnostic biomarker in liquid‐based urine cytology. hTERT was exclusively overexpressed in urothelial carcinoma including SHGUC and HGUC samples (*n* = 176/181, 97.2%), as opposed to its low expression in NHGUC samples (*n* = 2/87, 2.3%). Since an increased activity of hTERT, which is a catalytic component of the human telomerase, is detected in human cancer but not in most normal cells, this enzyme has been considered as a promising tumor marker in various cancers, including urothelial carcinoma.^11^, ^24^ We adopted a specific hTERT antibody, SCD‐A7, which is the first FDA‐approved in vitro diagnostic test for assessing hTERT protein expression in urine cytology. Two previously published studies using the SCD‐A7 antibody on urine cytology also demonstrated increased hTERT expression rates in urothelial carcinoma. Allison et al.^24^ demonstrated that nine of the 11 HGUC samples showed hTERT positivity, while in another study by Xing et al.^25^ six of the nine SGHUC and HGUC samples showed strong staining with hTERT. These consistent findings support hTERT ICC utility in detecting malignant urine cytology samples. Our study enrolled a large cohort of urothelial malignancies, as 247/337 cases were histologically confirmed as carcinomas. hTERT ICC showed strong immunoreactivity not only in the cytology cases proved to be high‐grade urothelial carcinomas (*n* = 208/217, 95.9%) or CIS (*n* = 24/25, 96%) but also in the ones subsequently diagnosed as low‐grade urothelial carcinomas (*n* = 4/4, 100%) during follow‐up. Of interest, 92.2% (*n* = 59/64) of the AUC cytology cases histologically diagnosed as urothelial carcinomas also showed positive hTERT ICC expression. These findings suggest SCD‐A7 ICC could be applied to triage indeterminant cytology cases, by identifying the ones harboring urothelial carcinoma.

In the initial application of ICC, however, there was a quality issue related to the immunoreactivity of hTERT. We noticed severe background staining in addition to overstaining of epithelial cells, regardless of several staining protocol alterations, including antibody incubation or retrieval time. After several trials, we found this technical issue was possibly caused due to the unsuitable type of glass slides we initially applied. Subsequently, the aforementioned background staining was significantly reduced by performing ICC on the New Silane III slides (Muto Pure Chemicals Co., Ltd.), compared with other types of coated slides. New Silane III slides exhibit stronger adhesive strength and fewer dyeing spots during immunostaining. Therefore, we believe these slides are appropriate for ICC, at least for the samples prepared using the SurePath technique.

In our study, hTERT nuclear or nonspecific cytoplasmic staining was also identified in benign urothelial cells, such as umbrella cells or parabasal cells, which confused interpretation in as many as 29 cases (29/337, 8.6%). However, the immunoreactivity for hTERT was weaker in benign cells compared with urothelial carcinoma, which is concordant with the findings of previous studies.^24^, ^25^ For instance, Xing et al.,^25^ who performed ICC on ThinPrep slides, recognized hTERT positivity in normal or nonurothelial cells, yet more often a stronger staining pattern in urothelial carcinoma. More specifically, whereas 37/60 (62%) of their NHGUC cases showed hTERT immunopositivity, just 8/37 (22%) exhibited a strong signal intensity. In our cohort, only 2/87 NHGUC cases (2.3%) exhibited strong hTERT immunopositivity. Therefore, when evaluating hTERT ICC, it is necessary to combine the intensity of hTERT immunoreactivity with cytomorphology. However, further studies would be necessary for a prospective validation of these findings, using different preparation methods (e.g., ThinPrep vs. SurePath) and sample types.

The current study is limited by its small number of atypical cytology samples that proved to be benign in histological follow‐up, also the exclusion of some cases due to insufficient cellularity or the lack of positive internal control during hTERT ICC. Therefore, future prospective studies should primarily focus on the AUC reporting category, including higher numbers of AUC cases associated with benign histology, to reflect better routine clinical practice and thus evaluate more efficiently the value of hTERT ICC in urine cytology. Nevertheless, our findings suggest that hTERT ICC using the SCD‐A7 antibody might be able to overcome the limitations of other methods, including genetic tests detecting hTERT promoter mutations or mRNA expression.^15^, ^16^, ^17^, ^26^ These genetic tests are costly and time‐consuming,^27^ while genetic expression is not necessarily associated with protein expression. Furthermore, the translated proteins themselves are the functional units within each cell in both physiological and pathological conditions.^21^, ^28^

In conclusion, the results of this study demonstrated that hTERT ICC appears to be a reliable biomarker for detecting malignancy in liquid‐based urine cytology. In addition, it might help triage cases showing AUC by identifying the ones harboring urothelial carcinoma.

## AUTHOR CONTRIBUTIONS

**Ji Hye Moon:** Conceptualization (equal); formal analysis (lead); investigation (equal); writing – original draft (lead). **Ilias P. Nikas:** Investigation (equal); writing – review and editing (equal). **Kyung Chul Moon:** Supervision (equal). **Bohyun Kim:** Investigation (equal). **Han Suk Ryu:** Conceptualization (equal); supervision (lead); writing – review and editing (lead).

## CONFLICT OF INTEREST STATEMENT

The authors declare no conflicts of interest.

## ETHICAL APPROVAL STATEMENT

The study protocol was reviewed and approved by the institutional review board of the Seoul National University Hospital (IRB no. 1907–183‐1051). The IRB also waived the requirement to obtain written informed consent from the patients.

## Data Availability

The data that support the findings of this study are available from the corresponding author upon reasonable request.
